# Disseminated Candidiasis and Candidemia Caused by *Candida palmioleophila* in a Green Sea Turtle (*Chelonia mydas*)

**DOI:** 10.3390/ani11123480

**Published:** 2021-12-07

**Authors:** Wen-Lin Wang, Pei-Lun Sun, Chi-Fei Kao, Wen-Ta Li, I-Jiunn Cheng, Pin-Huan Yu

**Affiliations:** 1 Institute of Veterinary Clinical Science, School of Veterinary Medicine, National Taiwan University, Taipei 10617, Taiwan; r06643010@ntu.edu.tw; 2 National Taiwan University Veterinary Hospital, National Taiwan University, Taipei 10672, Taiwan; 3 College of Medicine, Chang Gung University, Taoyuan 33302, Taiwan; sunfungus@gmail.com; 4 Research Laboratory of Medical Mycology, Department of Dermatology, Chang Gung Memorial Hospital, Linkou Branch, Taoyuan 33302, Taiwan; 5 Graduate Institute of Molecular and Comparative Pathology, School of Veterinary Medicine, National Taiwan University, Taipei 10617, Taiwan; fei81005@gmail.com; 6 Institute of Molecular Biology, Academia Sinica, Taipei 11529, Taiwan; 7 Pangolin International Biomedical Consultant Ltd., Keelung 20145, Taiwan; heerolee1104@gmail.com; 8 Institute of Marine Biology, National Taiwan Ocean University, Keelung 20224, Taiwan; b0107@mail.ntou.edu.tw

**Keywords:** *Candida palmioleophila*, candidemia, central venous catheter, *Chelonia mydas*, disseminated candidiasis, green sea turtle

## Abstract

**Simple Summary:**

A sub-adult green sea turtle was rescued and treated for shell fractures on the carapace and plastron. During the first 2 months, the turtle was kept dry-docked with a placement of an intravenous catheter. Pain management, antibiotic and anthelmintic therapy, fluid therapy, human albumin, force feeding, and wound debridement were provided to manage the shell fractures and to control the infection. After 2 months of care, small budding yeasts were noted on the turtle’s blood smears. Blood cultures yielded yeast-like colonies that were later identified as *Candida palmioleophila*. The patient was then treated with an antifungal agent and the catheter was removed. Approximately 3.5 months later, the carapace and plastron wounds had healed. However, the turtle died at 7.5 months after rescue. The postmortem examination revealed disseminated yeast in joints, bones, brain, and lungs. This study aims to improve the veterinary medical care and, therefore, enhance the conservation of endangered sea turtles by describing a rare report of systemic *C. palmioleophila* infection.

**Abstract:**

A sub-adult green sea turtle (*Chelonia mydas*) was rescued and treated for carapace and plastron shell fractures. The turtle was kept dry-docked for the first 2 months with a placement of a long-term jugular central venous catheter (CVC). Pain management, aggressive antibiotic and anthelmintic therapy, fluid therapy, force feeding, and wound debridement were provided to manage the shell fractures and control bacteremia. Human albumin was administered to treat severe hypoalbuminemia. On day 59, small budding yeasts were noted on the blood smears. Candidemia was confirmed by blood culture, as the yeasts were identified as *Candida palmioleophila* by the molecular multi-locus identification method. The CVC was removed, and the patient was treated with itraconazole. Although the carapace and plastron wounds had epithelized by 5.5 months after the rescue, the turtle died unexpectedly by 7.5 months. The postmortem examination revealed numerous necrogranulomas with intralesional yeasts, morphologically compatible with *Candida* spp., in joints, bones, brain, and lungs, suggestive of disseminated candidiasis. We describe a rare case of candidemia in the veterinary field. To our knowledge, this is the first report of candidiasis caused by *C. palmioleophila* in a reptile. The present results should improve veterinary medical care and, therefore, enhance the conservation of endangered sea turtle species.

## 1. Introduction

All sea turtle species are on the International Union for Conservation of Nature’s Red List of Threatened Species and are also listed as a protected species by the Taiwan Wild Animal Conservation Act. Veterinary medical management and pathology studies of diseased turtles are components of sea turtle conservation programs. In Northern Taiwan, the Marine Ecology and Conservation Laboratory at National Taiwan Ocean University has been dedicated to rescuing stranded sea turtles and investigating sea turtle mortality since 2007. In 2012, National Taiwan University Veterinary Hospital began cooperating with the lab to assist and provide professional veterinary medical care.

*Candida* is a ubiquitous fungus that can be readily isolated from the reptile gastrointestinal tract, skin, and respiratory system. Numerous *Candida* species have been isolated from oral, cloacal, and skin swabs of healthy reptiles. As such, *Candida* species usually cause opportunistic gastrointestinal, cutaneous, or systemic infections in stressed or immunosuppressed reptiles [1]. Although candidiasis is not uncommon in reptiles, reports of invasive candidiasis in reptiles are scarce [2,3]. Moreover, to date, there have been no confirmed reports in the veterinary literature of candidemia in a reptile.

We report here the clinical case of a sub-adult green sea turtle with disseminated candidiasis and candidemia caused by *Candida palmioleophila*, possibly associated with infected external wounds or intravenous line. The candidemia was confirmed antemortem by peripheral blood smears and blood culture. This study aims to improve veterinary medical care and, therefore, enhance the conservation of an endangered sea turtle species by identifying and characterizing this rare species of *Candida* via detailed descriptions of the clinical, histopathological, and mycological features.

## 2. Materials and Methods

### 2.1. Case

A female green sea turtle (*Chelonia mydas*) weighing 31.3 kg was found stuck inside a cold seawater intake drain of the Tunghsiao gas-fired power plant in Miaoli County (decimal degrees: 24.49127, 120.67422), Taiwan, on 4 September 2018. The turtle’s curved carapace length measured 74.2 cm and the curved carapace width measured 65.7 cm, indicating that the turtle was sub-adult [4]. The body condition index ([weight (kg)/straight carapace length (cm^3^)] × 10,000) was 0.77, indicating that the turtle was emaciated [4]. The turtle had multiple shell fractures on the carapace and plastron. The turtle was immediately rescued by Taiwan Sea Turtle Conservation Society, and honey was applied to all of the affected surfaces before the turtle arrived at National Taiwan University Veterinary Hospital.

On physical examination, the turtle was lethargic but responsive to outer stimuli. Shell fractures on both the carapace and plastron were noted. Scattered barnacles were present above the carapace and removed manually. The remainder of the physical examination findings and neurological examination findings were unremarkable, and no other significant abnormalities were identified. Hematologic testing, plasma biochemical analysis, plasma protein electrophoresis, radiography, and computed tomography (CT) were performed.

Blood was collected from the external jugular vein (dorsal cervical sinus). The results of hematologic testing showed moderate heterophilia (57%; reference interval, 8–35%) with toxic heterophils (3+ toxicity) [4,5]. The results of biochemical analyses showed low total calcium (Ca) concentration (6.2 mg/dL; reference interval, 8.0–8.8 mg/dL) with normal ionized Ca (1.015 mmol/L; reference interval, 0.57–1.06 mmol/L), hypocholesterolemia (93 mg/dL; reference interval, 142–354 mg/dL), hypotriglyceridemia (45 mg/dL; reference interval, 124–932 mg/dL), increased aspartate aminotransferase level (323 U/L; reference interval, 74–245 U/L), elevated lactate dehydrogenase level (2601 U/L; reference interval, 75–477 U/L), and elevated creatine kinase concentration (8720 U/L; reference interval, 326–2729 U/L) [6,7]. Plasma protein electrophoresis showed a low albumin–globulin ratio of 0.22 (reference interval, 0.42–1.19) [8]. The turtle had a severe infection and inflammatory status with muscle damage and malnutrition.

Survey radiographs (dorsoventral view, right lateral view, and anterior-posterior view) showed only moderate gastrointestinal gas; however, the severity and depth of the shell fractures were vague. The CT scans (Activion 16, Toshiba Corp., Tokyo, Japan) were performed without contrast and revealed that multiple linear fractures presented from the left nuchal plate, crossed the midline, and extended to the right central area of the carapace. Several right transverse processes of the vertebrae were also completely fractured beneath the trauma area.

The turtle was then anesthetized using 10 mg/kg intravenous (IV) propofol (Propofol^®^ Lipuro 1%, 10 mg/mL, B. Braun Melsungen AG, Berlin, Germany) for the placement of a long-term jugular polyurethane central venous catheter (CVC) (CAREFLOW™ 2 Lumen Central Venous Catheter, Argon Medical Devices, Singapore) using the modified Seldinger technique, as described previously [9]. After CVC placement, the patient was initially treated with butorphanol 0.5 mg/kg IV q24 h for 2 weeks (Torbugesic^®^ 10 mg/mL, Zoetis, Kalamazoo, MI, USA) and intramuscular (IM) meloxicam 0.2 mg/kg q24 h (Mobic^®^ inj. 15 mg/1.5 mL, Boehringer Ingelheim, Ingelheim am Rhein, Germany) for pain management, and amikacin 5 mg/kg IM q48 h for 2 weeks (Amikacin injection 125 mg/mL, Nang Kuang Pharmaceutical Co., Ltd., Tainan, Taiwan) as antibiotic therapy. Reptile ringer’s (1 part Lactated ringer’s with 2 parts 2.5% dextrose and 0.45% sodium chloride [YF Chemical Corp., New Taipei, Taiwan]) was administered at 1.25 mL/kg/h IV for supportive care. Praziquantel 25 mg/kg orally (PO) q3 h for 3 doses (Kaicide Tab 600-mg, Synpac-Kingdom Pharmaceutical Co., Ltd., Taipei, Taiwan) was administered prophylactically.

**Figure 1:**
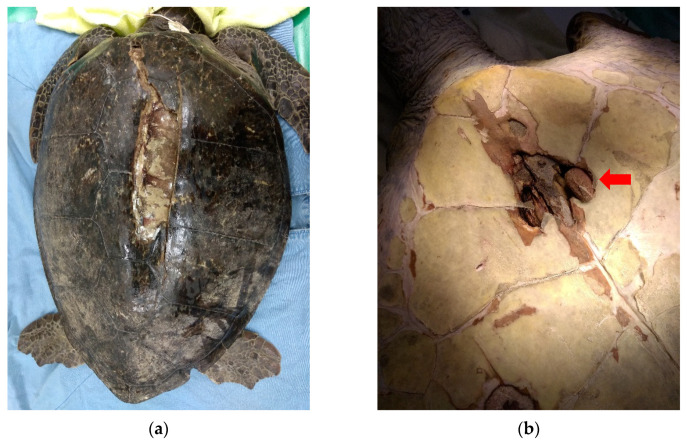
(**a**) After initial wound debridement, a large carapace defect with lung exposure was observed. (**b**) The left acromion process of the pectoral girdle (red arrow) was visible through the plastron defects located at the gular scutes and exhibited severe osteomyelitis with the presence of abundant pus.

Upon initial wound debridement, the patient was anesthetized with 10 mg/kg IV propofol (Propofol^®^ Lipuro 1%, 10 mg/mL, B. Braun Melsungen AG, Berlin, Germany). The whole shell was cleaned and scrubbed with chlorhexidine, then the affected shell areas on the carapace and plastron were irrigated and flushed with copious amounts of warm sterile saline solution. Debridement of wounds was performed using strictly aseptic techniques. Infected or necrotic tissues, debris, and unstable shell pieces were carefully removed, resulting in a large carapace defect with lung exposure (Figure 1a). The dead space under the cranial part of the carapace was so wide it made the removal of infected and necrotic tissues difficult. The left acromion process of the pectoral girdle was visible through the plastron defects located at the gular scutes and had severe osteomyelitis with the presence of abundant pus (Figure 1b). The infected bone was removed using a rongeurs. The fracture wounds were dressed with a mixture of honey and silver sulfadiazine (Anco cream (silver sulfadiazine), Royal Chemical & Pharmaceutical Co., Ltd., Kaohsiung, Taiwan), followed by an application of a wet-to-dry bandage with gauze pads and finally an application of OPsite^®^ (28 cm W × 30 cm L; Smith & Nephew, Hull, UK) to the outer layer. The wounds were debrided and irrigated and the bandages were changed daily during the first month. A sample of the infected bone obtained during debridement was submitted for aerobic and anaerobic bacterial culture, which revealed growth of *Shewanella putrefaciens* and *Morganella morganii*. Based on the sensitivity profiles of these organisms, the antibiotic was changed to trimethoprim/sulfamethoxazole 30 mg/kg IM q48 h (Sevatrim Injection Sulfamethoxazole 400 mg, Trimethoprim 80 mg/5 mL/amp, Swiss Pharmaceutical Co., Ltd., Tainan, Taiwan).

During hospitalization, the turtle was kept dry-docked during the first 2 months and remained anorexic during the entire hospitalization period. Force feeding (Oxbow^®^ Critical Care, Emeraid^®^ Intensive Care, squid, shrimp, seaweed) of 20 mL/kg of body weight was provided every 3 days.

Follow-up blood examinations (complete blood count, blood smear, and biochemistry) were performed weekly for monitoring the turtle’s condition. Bacterial cultures either from the wounds or blood were performed monthly for antibiotic selection. On day 21 (25 September), bacteremia was detected via the blood smear, so blood obtained from the CVC contralateral-side dorsal cervical sinus was sent for aerobic bacterial culture (using BD BACTEC™ Peds Plus™/F vial). Aerobic bacterial blood culture results showed growth of *Klebsiella pneumoniae* and *M. morganii*. Based on the sensitivity profiles, the antibiotic was changed to ciprofloxacin 10 mg/kg IV q48 h (Ciproxin infu sol 200 mg/100 mL, Bayer, Germany) combined with metronidazole 20 mg/kg IV q24 h (Metronidazol 500 mg/100 mL/bot, Fresenius Kabi, Germany). At this time, itraconazole 15 mg/kg PO q72 h (itrazole oral soln 10 mg/mL, Hwangs Pharmaceutical Co., Ltd., Yunlin, Taiwan) was also administered prophylactically to prevent secondary fungal infection in this immunocompromised turtle. The state of immunocompromise was a collective conclusion based on the stress related to long-term dry docking and severe infection. Human albumin 150 mL (U.S.P. Albutein 25%, Grifols Biologicals Inc., Los Angeles, CA, USA) was diluted with 600 mL of 0.9% saline and provided at 0.8 mL/kg/h IV to treat hypoalbuminemia (Albumin 0.42 g/dL with low albumin–globulin ratio of 0.14) on day 37 (11 October). On the same day, the turtle was anesthetized with dexmedetomidine 0.1 mg/kg IM (DEXDOMITOR^®^, Zoetis, Kalamazoo, MI, USA), midazolam 1 mg/kg IM (Dormicum^®^ 5 mg/mL, Roche, Basle, Switzerland), ketamine 5 mg/kg IM (Imalgène 1000 100 mg/mL, Merial, Lyon, France), and propofol 2 mg/kg IV for shell fracture fixation surgery using screws and wires because the wound was determined as having no obvious sign of infection. The infected left acromion process of the pectoral girdle was sampled again for aerobic bacterial culture. Culture results showed growth of multidrug-resistant *Enterobacter cloacae*, *Klebsiella pneumonia*, *Aeromonas hydrophila*, and *M. morganii*. Based on the sensitivity profiles, the antibiotic was changed to meropenem 20 mg/kg IV q12 h (Meropenem 500 mg, Sandoz, Holzkirchen, Germany) combined with metronidazole 20 mg/kg IV q24 h for 2 weeks to continue controlling the severe systemic infection and bacteremia. On day 41 (15 October), albumin was elevated to 0.92 g/dL with an increased albumin–globulin ratio of 0.31.

**Figure 2:**
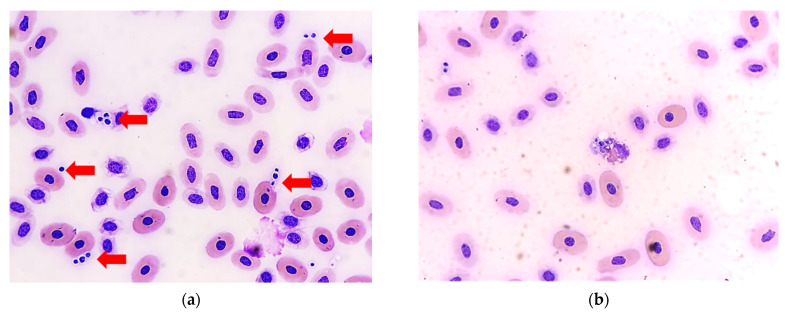
(**a**) Groups of round- to oval-shaped yeast cells were observed in the blood smear of the sea turtle (red arrow). (**b**) Some yeast cells were ingested by a heterophil (Wright-Giemsa stain, 400× original magnification).

On day 59 (5 November), numerous small budding yeasts were noted on blood smears, some of which were ingested by heterophils (Figure 2). The CVC was removed (indwelling time from day 23 to day 59 (37 days total)) without replacement. Meanwhile, a second aerobic bacterial blood culture was performed, which yielded yeast-like colonies and *Enterococcus faecalis*. The yeast-like colonies were picked and purified by subculture on Sabouraud’s dextrose agar and subjected to further mycological testing.

We continued to administer itraconazole 15 mg/kg PO q72 h and the antibiotic was changed to piperacillin 50 mg/kg IM q24 h for 4 weeks (Piperacillin powder 2 gm/vial, Chunghwa Yuming Healthcare Co., Ltd., Taipei, Taiwan) based on sensitivity testing results. After 1 week, no yeasts or bacteria were found on the blood smears, and the WBC count decreased from 11,544/μL to 9944/μL. Itraconazole was maintained for 1 month.

The turtle was discharged and cared for by Marine Ecology and Conservation Laboratory at National Taiwan Ocean University on day 113 (26 December) after the systemic infection was presumably resolved and the blood examination was almost normal, with only hypoalbuminemia remaining. The turtle began to eat by itself on day 125 (7 January 2019). The carapace and plastron wounds had fully epithelized by day 167 (26 February). Unfortunately, the turtle was unexpectedly found dead on day 233 (25 April) and submitted for postmortem evaluation. Before the turtle died, the mobility of the limbs was normal.

### 2.2. Mycological Studies

The yeast isolate was sent to the Research Laboratory of Medical Mycology, Linkou Chang Gung Memorial Hospital, for further identification and deposited under strain number CGMHD 2210. The isolate was inoculated on potato dextrose agar and incubated at 25 °C.

For molecular identification, the internal transcribed spacer regions of ribosomal DNA (*ITS*), partial large subunit of 28S ribosomal DNA (*LSU*), partial transcription elongation factor 1-alpha (*TEF1a*) gene, and partial RNA polymerase II largest subunit (*RPB1*) gene were amplified and sequenced. The primers used were ITS1: TCCGTAGGTGAACCTGCGG and ITS-4: TCCTCCGCTTATTGATATGC (for *ITS*), NL-1: GCATATCAATAAGCGGAGGAAAAG and NL-4: GGTCCGTGTTTCAAGACGG (for *LSU*), EF1-983F: GCYCCYGGHCAYCGTGAYTTYAT and EF1-2218R: ATGACACCRACRGCRACRGTYTG (for *TEF1a*), and RPB1-Af: GARTGYCCDGGDCAYTTYGG and RPB1-Cr: CCNGCDATNTCRTTRTCCATRTA (for *RPB1*). The PCR products were purified and then sequenced using an ABI Prism model 3730 × 1 DNA Analyzer^®^ (Applied Biosystems, Foster City, CA, USA).

### 2.3. Antifungal Susceptibility Testing

The isolate was subjected to antifungal susceptibility testing using Sensititre^™^ YeastOne^™^ (Thermo Scientific, Pittsburgh, PA, USA), according to the manufacturer’s protocol.

### 2.4. Postmortem Examination and Histopathology

The postmortem examination was performed by two experienced veterinary pathologists (C.F.K. and W.T.L.). Representative tissue samples were collected from the brain, thyroid glands, trachea, lungs, heart, gastrointestinal tract, spleen, liver, both adrenal glands, and urogenital tract. The samples were fixed in 10% neutral-buffered formalin, processed routinely, and stained with hematoxylin and eosin.

## 3. Results

### 3.1. Mycological Studies

**Figure 3:**
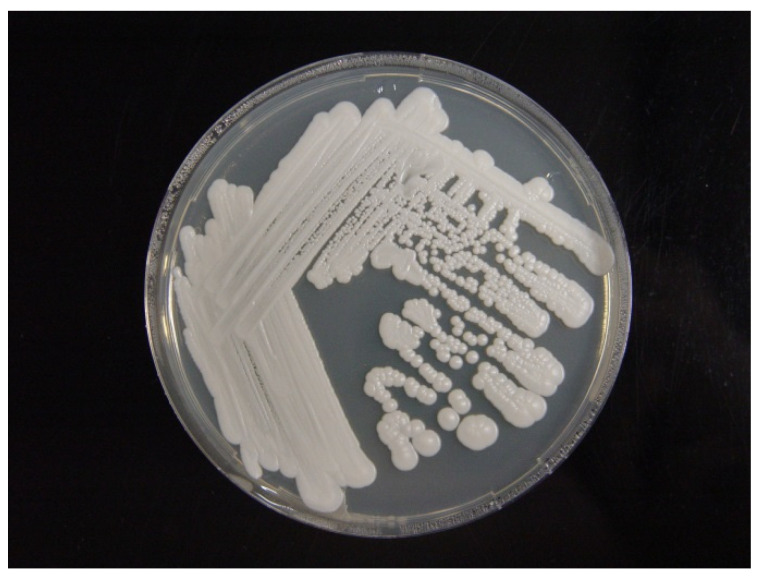
Culture of *C. palmioleophila* isolated from the blood and grown on potato dextrose agar at 25 °C for 14 days.

**Figure 4:**
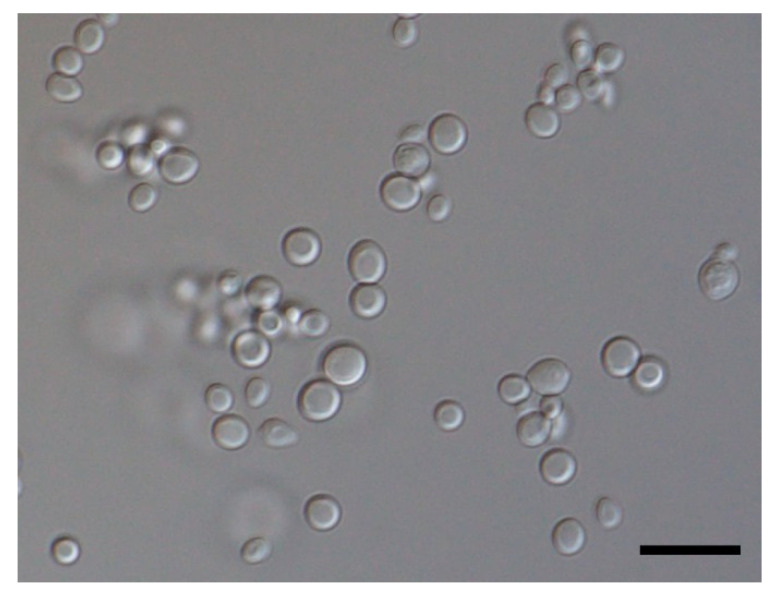
Micrograph showing isolated cells of the yeast *C. palmioleophila* (scale bar = 10 µm).

The isolate grew well on potato dextrose agar at 25 °C (Figure 3). A wet preparation of the isolate showed single yeast cells of variable sizes, measuring 2.42–5.29 × 2.04–4.09 µm (Figure 4). No pseudohyphae were noted after 14 days of culturing.

**Figure 5:**
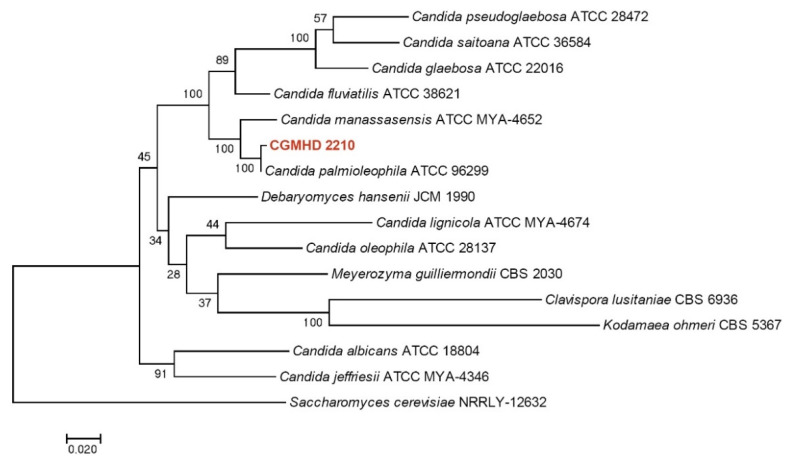
Maximum-likelihood phylogenetic tree based on combined *ITS-LSU-TEF1a-RPB1* sequences. The Tamura 3-parameter model was used as the substitutional model. A discrete gamma distribution was used to model evolutionary rate differences between sites. The rate variation model allowed for some sites to be evolutionarily invariable. Bootstrap values are shown at the nodes. The scale bar indicates the number of substitutions per site. *Saccharomyces cerevisiae* was used as an outgroup.

The sequence similarity between CGMHD 2210 and *C. palmioleophila* type strain ATCC 96299 was 100% for *ITS* and *LSU*, 99% for *TEF1a*, and 98% for *RPB1*. Figure 5 shows a phylogenetic tree constructed based on maximum-likelihood analysis using combined data sets for the *ITS*, *LSU*, *TEF1a*, and *RPB1* sequences of CGMHD 2210 and selected species of *Candida* and related genera. MEGA 7 software [10] was used for the analysis. Sequences used in this analysis are listed in Table 1. The isolate from the current turtle was in the same clade as *C. palmioleophila* and exhibited a good supportive value.

### 3.2. Antifungal Susceptibility Testing

The minimal inhibitory concentrations of nine antifungal agents against the isolate were as follows: amphotericin B = 0.5 µg/mL, flucytosine = 0.12 µg/mL, fluconazole = 16 µg/mL, itraconazole = 0.5 µg/mL, voriconazole = 0.25 µg/mL, posaconazole = 0.25 µg/mL, caspofungin = 0.12 µg/mL, micafungin = 0.06 µg/mL, anidulafungin = 0.03 µg/mL.

### 3.3. Pathological Examination

**Figure 6:**
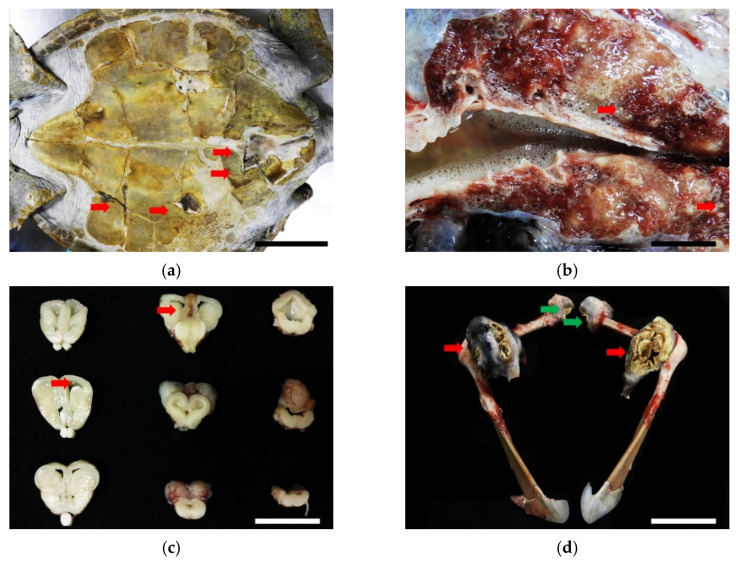

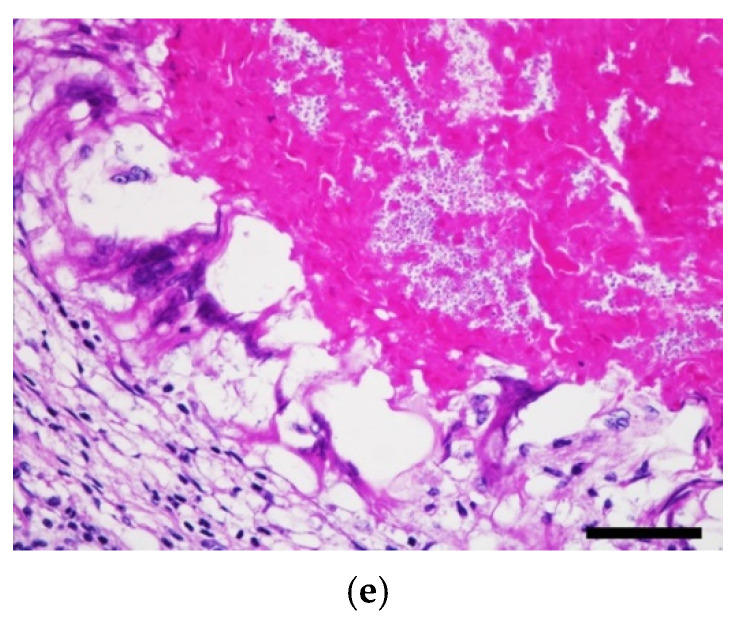
(**a**) Four irregular- and variably-sized puncture wounds were observed on the gular, humeral, pectoral, and femoral scutes of the plastron (red arrows). Scale bar = 15 cm. (**b**) Multiple, 0.2 × 0.2 × 0.2 cm, irregular, beige to yellowish nodules were noted on the cut sections of lungs (red arrows). Scale bar = 2 cm. (**c**) The same nodules were noted on serial sections of the brain (red arrows). Scale bar = 2.5 cm. (**d**) Both acromion processes were deformed and replaced by approximately 8 × 5 × 5 cm encapsulated masses consisting of fragile caseous materials (red arrows). The articular surfaces of both humeroradial joints were rough with caseous materials (green arrows). Scale bar = 10 cm. (**e**) In the neuroparenchyma, necrogranulomas containing numerous round-to-oval, 2 to 4 μm-diameter, pale-staining, thin-walled yeasts were noted. Hematoxylin and eosin stain. Scale bar = 50 μm.

The postmortem examination revealed a 15 × 5 cm-large, triangular open wound in addition to fractures affecting the vertebral scutes of the carapace with granulation tissue formation. Four irregular- and variably-sized puncture wounds were observed at the gular, humeral, pectoral, and femoral scutes of the plastron (Figure 6a). The above-mentioned wounds penetrated into the subjacent soft tissues and bones, with caseous materials. The trachea and bronchi were filled with frothy fluid and both lung lobes were wet and heavy. On cut sections of the lungs and brain, multiple 0.2 × 0.2 × 0.2 cm, irregularly shaped, beige to yellowish nodules were noted (Figure 6b,c). The bilateral acromion processes and left ilium process were deformed and, respectively, replaced by approximately 8 × 5 × 5 cm and 5 × 5 × 5 cm encapsulated masses containing fragile caseous materials. The articular surfaces of both humeroradial joints and the left hip joint were rough with caseous materials (Figure 6d). There were no additional macroscopic lesions noted in other organs.

Histopathologically, numerous variably-sized necrogranulomas multi-focally affecting the pulmonary interstitium, neuroparenchyma, and ventricles were observed and formed a core of brightly eosinophilic and karyorrhectic debris surrounded by numerous multinucleated giant cells with fewer lymphocytes, plasma cells, and rare heterophils with delicate fibrous connective tissue. Numerous round-to-oval, 2 to 4 μm-diameter, pale-staining, and thin-walled yeasts were observed in the necrogranulomas (Figure 6e). The caseous lesions of the joints and bones contained abundant hypereosinophilic necrotic debris with mixed fungal and bacterial populations and were encapsulated by a thick layer of fibrous connective tissue, suggestive of necrogranulomatous polyarthritis and osteomyelitis. In addition to the fungus-associated necrogranulomatous, lower numbers of necrogranulomas containing mixed-bacterial populations were detected in the lungs, liver, hepatic veins, intestines, and kidneys. No acid-fast-positive organisms were observed in these necrogranulomas.

## 4. Discussion

*Candida palmioleophila* is an uncommon *Candida* species and therefore easily misidentified [11]. It was first described in 1988 by Nakase et al. based on a strain isolated from soil [12]. *Candida palmioleophila* are single yeast cells that do not form pseudohyphae in culture and can tolerate high temperatures, up to 42 °C. Due to similar phenotypic characteristics, misidentification of *C. palmioleophila* as *C. famata* (*Debaryomyces hansenii*) or *C. guilliermondii* (*Pichia guilliermondii*) is not uncommon [13]. However, *C. palmioleophila* can be unambiguously distinguished from other similar species using molecular methods such as DNA sequencing [11]. *Candida palmioleophila* has a molecular sister species, *C. manassasensis*, in the *C. glaebosa* clade. These two species have similar ITS and D1/D2 regions within the 28S ribosomal DNA sequences that are commonly used for fungal identification. However, they can be discriminated based on the sequences of their elongation factor-1α gene (*TEF-1α*), RNA polymerase II largest subunit gene (*RPB1*), and second largest RNA polymerase II subunit gene (*RPB2*) [14].

Based on limited published data, *C. manassasensis* appears to exhibit high minimal inhibitory concentration values for fluconazole, intermediate–low values for voriconazole and itraconazole, and low values for echinocandins [11,15,16]. Our isolate exhibited a susceptibility profile similar to those previously reported. Although there are currently no clinical breakpoints of antifungals against *C. palmioleophila*, a correct diagnosis is essential in clinical settings because of possible innate resistance of this *Candida* species to fluconazole.

*Candida palmioleophila* has been isolated from the mouth cavity of a wild South American river turtle (*Podocnemis expansa*) [17], the skin and shell of healthy aquatic turtles [1], scarlet ibises (*Eudocimus ruber*) in Brazil [15], and healthy turkeys in Poland [18]. This *Candida* species has also caused catheter-related fungemia in humans [19].

Invasive candidiasis, including disseminated/systemic candidiasis and candidemia, is a severe *Candida* infection affecting multiple organs and exhibiting a wide hematogenous spread. The infection may originate from any organ, such as the gastrointestinal tract, or from infected intravenous lines [20]. In our present case, it is difficult to determine the exact source and route of the systemic *Candida* infection. Infection either from catheterization or the deep external wounds causing candidemia and then polyarthritis/osteomyelitis is possible.

Candidiasis is not uncommon in reptiles. Previous reports of *Candida* spp. infection in reptiles are summarized in Table 2, with gastrointestinal candidiasis as the most common. However, reports of invasive candidiasis in reptiles are rare, with only one report of *C. albicans*-disseminated infection in an elongated tortoise (*Indotestudo elongata*) [2] and one report of systemic *Candida* spp. infection originating from deep gastric mycosis in a wild Indian crocodile [3]. To date, successful diagnosis of invasive candidiasis in reptiles is difficult, and effective treatments are lacking.

Although postmortem fungal culture was not performed in our case, the yeasts found in the central nervous system, lungs, joints, and bones were indicative of a disseminated fungal infection. The histological findings, in conjunction with the results of antemortem blood cultures, are supportive of candidemia caused by *C. palmioleophila* with secondary disseminated candidiasis. Furthermore, necrogranulomatous lesions with mixed bacterial clumps in multiple organs, including the lungs, liver, hepatic veins, intestines, kidneys, joints, and bones, could have resulted from systemic bacterial infection secondary to the external trauma.

Candidemia is the most common type of fungemia in humans and it is strongly associated with CVC placement and immunocompromised patients [27]. Certain species, such as *C. parapsilosis*, are strongly associated with CVC-related candidemia [28]. Although a CVC might stay in place without any complications for months in chelonians with proper care [9], regular monitoring for potential complications, such as infection, phlebitis, hematoma, artery or nerve damage, or subcutaneous extravasation, is warranted.

Some risk factors for candidemia have been identified in humans, including long length of hospital stay, abdominal surgery, femoral artery catheterization, blood transfusions, parenteral nutrition, and previous use of meropenem or other broad-spectrum antibiotics [29,30,31]. Our case involved the use of several antibiotics based on the results of bacterial cultures and sensitivity tests, including meropenem, to control the serious systemic infection. Human albumin was given to counter the severe hypoalbuminemia, and the turtle was hospitalized for 2 months prior to detection of candidemia; these factors might have increased the risk of infection. Although ineffective and prolonged broad-spectrum antibiotic treatment in chelonians has been determined to increase the risk of opportunistic infections with *Candida* [2], the actual risk factors in reptiles still need further investigation.

The gold-standard diagnostic tool for candidemia is blood culture; however, the infection can sometimes be detected via blood smears if the disease is extremely progressive [32,33]. Treatments are well-studied in human medicine and include antifungal and early catheter removal, whether or not the candidemia is catheter-related [28]. Early removal of the catheter might reduce the risk of mortality from candidemia [34]. Treatment duration depends on daily blood culture results and should be continued for 2 weeks after blood cultures become negative [35].

There are several flaws in our present case. First, we did not repeat the blood culture after starting the treatment. Although no *Candida* was found on the blood smears 1 week after removing the CVC, it is essential to perform follow-up blood cultures to confirm that the pathogen has been cleared, because the sensitivity of blood smears to detect candidemia is relatively low [32]. Second, the uncontrollable infection due to multidrug-resistant bacteria was related to the immunocompromised condition caused by long-term stress related to dry-docking and other medical procedures. The balance between adequate medical care and the animal’s stress should be carefully measured. Additionally, the use of aggressive antibiotics is vital to treat the bacteremia in this animal, however, this might be unnecessary. This may lead to opportunistic fungal infections. According to the antibiotic stewardship for reptiles [36], veterinarians should use antibiotics conditionally and third-line antibiotics (e.g., carbapenems) should generally not be used in veterinary medicine. Third, the cause of death of the sea turtle could be a mix of both *candida* infection and bacterial infection. The failure of the treatment may be attributed to impediments to the systemic antimicrobial drugs reaching the joints and bones. The considerable amount of caseous material observed could have served as a haven for *Candida* spp. and other bacteria, allowing them to escape from systemic antimicrobial drugs and become the source for later disseminated diseases. In addition, postmortem bacterial and fungal culture should be conducted to compare with the results of antemortem microbial cultures. Lastly, with the patient showing no signs of reduced limb mobility or weakness of the affected limb, we also failed to detect the polyarthritis clinically. Polyarthritis and osteomyelitis are common in sea turtles with immunosuppressive status (caused by trauma, malnutrition, or stress) [37]. They are usually bacterial [37,38,39,40,41,42] and rarely concurrently fungal [43]. Therefore, regular follow-up radiographs are important for sea turtles, especially those undergoing frequent handling.

## 5. Conclusions

We described a rare case of disseminated candidiasis and candidemia in the veterinary field. To the best of our knowledge, this is the first report of *C. palmioleophila* systemic infection in a reptile. We also successfully diagnosed invasive candidiasis by antemortem blood smears and blood culture. The current report also provides detailed information regarding the clinical, histopathological, and mycological characteristics of *C. palmioleophila*. With the increasing awareness of fungal pathogens and related diseases in the One Health concept, this information doubtlessly contributes to the further understanding of a limited-reported fungal species.

Although the turtle did not survive, it cannot be doubted that the veterinary critical care and applied medical procedures helped the turtle recover from its severe wounds and debilitated state. Veterinary medicine not only provides medical care, it also aids in the conservation of endangered sea turtles.

## Figures and Tables

**Table 1:** Sequences used to construct the phylogenetic tree.

Species Name	Strain Number	*ITS*	*LSU*	*TEF1α*	*RPB1*
Current isolate	CGMHD 2210	MZ317464	MZ317465	MZ328223	MZ328224
*Candida albicans*	ATCC 18804	HQ876043	HQ876051	KC507436	KC507405
*Candida fluviatilis*	ATCC 38621	HQ652068	KC479696	KC507441	KC507410
*Candida glaebosa*	ATCC 22016	KC479686	KC479693	KC507437	KC507406
*Candida jeffriesii*	ATCC MYA-4346	FJ623615	FJ614682	KC507445	KC507414
*Candida lignicola*	ATCC MYA-4674	HQ652074	HQ652029	KC507469	KC507427
*Candida manassasensis*	ATCC MYA-4652	HQ652050	HQ651971	KC507463	KC507423
*Candida oleophila*	ATCC 28137	HQ876045	HQ876053	KC507438	KC507407
*Candida palmioleophila*	ATCC 96299	KC479687	KC479697	KC507449	KC507418
*Candida pseudoglaebosa*	ATCC 28472	HQ652066	KC479694	KC507439	KC507408
*Candida saitoana*	ATCC 36584	HQ652067	KC479695	KC507440	KC507409
*Clavispora lusitaniae*	NRRL Y-11827	NR130677	JQ689030	JQ699057	JQ713040
*Debaryomyces hansenii*	NRRL Y-7426	NR120016	JQ689041	JQ699068	JQ713052
*Kodamaea ohmeri*	NRRL Y-1932	NR121464	GU597323	GU597338	JQ713038
*Meyerozyma guilliermondii*	NRRL Y-2075	NR111247	JQ689047	JQ699074	JQ713058
*Saccharomyces cerevisiae*	NRRL Y-12632	AY046146	JQ689017	JQ699041	JQ713023

**Table 2:** Reported cases of *Candida* spp. infection in reptiles.

Species	Reptile Species Affected	Reference
*C. albicans*	Crocodile tegu (*Crocodilurus lacertinus*)	[21]
	Radiated tortoise (*Geochelone radiata*)	[21]
	Smooth snake (*Coronella austriaca*)	[21]
	Loggerhead sea turtle (*Caretta caretta*)	[22]
	Greek tortoise (*Testudo graeca*)	[23]
	Elongated tortoise (*Indotestudo elongata*)	[2]
*C. guilliermondii*	Fischer’s chameleon (*Chamaeleo fischeri*)	[24]
	Jackson’s chameleon (*Chamaeleo jacksoni*)	[24]
*C. palmioleophila*	Green sea turtle (*Chelonia mydas*)	This case
*C. tropicalis*	Hermann’s tortoise (*Testudo hermanni*)	[25]
*Candida* species	Mugger crocodile (*Crocodylus palustris*)	[3]
	Aldabra Giant Tortoise (*Geochelone gigantea*)	[26]

## Data Availability

The data presented in this study are available on request from the corresponding author.
